# Biallelic mutations in *CYP24A1* or *SLC34A1* as a cause of infantile idiopathic hypercalcemia (IIH) with vitamin D hypersensitivity: molecular study of 11 historical IIH cases

**DOI:** 10.1007/s13353-017-0397-2

**Published:** 2017-05-03

**Authors:** Ewa Pronicka, Elżbieta Ciara, Paulina Halat, Agnieszka Janiec, Marek Wójcik, Elżbieta Rowińska, Dariusz Rokicki, Paweł Płudowski, Ewa Wojciechowska, Aldona Wierzbicka, Janusz B. Książyk, Agnieszka Jacoszek, Martin Konrad, Karl P. Schlingmann, Mieczysław Litwin

**Affiliations:** 1 Department of Pediatrics, Nutrition and Metabolic Diseases, The Children’s Memorial Health Institute, Aleja Dzieci Polskich 20, 04-730 Warsaw, Poland; 2 Department of Medical Genetics, The Children’s Memorial Health Institute, Warsaw, Poland; 3 Department of Biochemistry, Radioimmunology and Experimental Medicine, The Children’s Memorial Health Institute, Warsaw, Poland; 4 Department of Nephrology, The Children’s Memorial Health Institute, Warsaw, Poland; 5 Department of Medical Genetics, Warsaw Medical University, Warsaw, Poland; 6 Postgraduate School of Molecular Medicine, Warsaw, Poland; 7 University Children’s Hospital, Münster, Germany

**Keywords:** Idiopathic infantile hypercalcemia, Vitamin D hypersensitivity, Biallelic mutations, *CYP24A1*, *SLC34A1*, Adults

## Abstract

Idiopathic infantile hypercalcemia (IIH) is a mineral metabolism disorder characterized by severe hypercalcemia, failure to thrive, vomiting, dehydration, and nephrocalcinosis. The periodical increase in incidence of IIH, which occurred in the twentieth century in the United Kingdom, Poland, and West Germany, turned out to be a side effect of rickets over-prophylaxis. It was recently discovered that the condition is linked to two genes, *CYP24A1* and *SLC34A1*. The aim of the study was to search for pathogenic variants of the genes in adult persons who were shortlisted in infancy as IIH caused by “hypersensitivity to vit. D”. All persons were found to carry mutations in *CYP24A1* or *SLC34A1*, nine and two persons respectively. The changes were biallelic, with one exception. Incidence of IIH in Polish population estimated on the basis of allele frequency of recurrent p.R396W *CYP24A1* variant, is 1:32,465 births. It indicates that at least a thousand homozygotes and compound heterozygotes with risk of IIH live in the country. Differences in mechanism of developing hypercalcemia indicate that its prevention may vary in both IIH defects. Theoretically, vit. D restriction is a first indication for *CYP24A1* defect (which disturbs 1,25(OH)_2_D degradation) and phosphate supplementation for *SLC34A1* defect (which impairs renal phosphate transport). In conclusion, we suggest that molecular testing for *CYP24A1* and *SLC34A1* mutations should be performed in each case of idiopathic hypercalcemia/hypercalciuria, both in children and adults, to determine the proper way for acute treatment and complications prevention.

## Introduction

Idiopathic infantile hypercalcemia (IIH) first received attention in the 1950s in the UK because of a sudden increase in the incidence. A number of years passed until it turned out to be a side effect of a just started program of rickets prophylaxis. Reducing vitamin D (vit. D) food supplementation normalized the situation (Samuel 1964).

We experienced a similar “endemic” condition in the 1970s in Poland (Pronicka et al. 1985), in the period of commonly administered so called “periodic” vit. D dosage (300,000 units, 3 times per year). The same phenomenon was observed in Eastern Germany (Misselwitz et al. 1990). At least 36 infants with IIH from the entire country were treated in our pediatric reference centre (Pronicka et al. 1985, 1988, 1997; Rowińska et al. 1998). During the normocalcemic phase of the disease, on continuous vit. D restriction, we found relatively high 25-hydroxyvitamin D (25OHD) and 1,25-dihydroxyvitamin D (1,25(OH)_2_D) concentrations. The condition was named “hypersensitivity to vitamin D”, to distinguish it from Williams syndrome and from other, non-vitamin D sensitive IIH types (Pronicka et al. 1988, 1997). Autosomal recessive inheritance was anticipated, with frequency about 1:60,000 births.

**Fig. 1:**
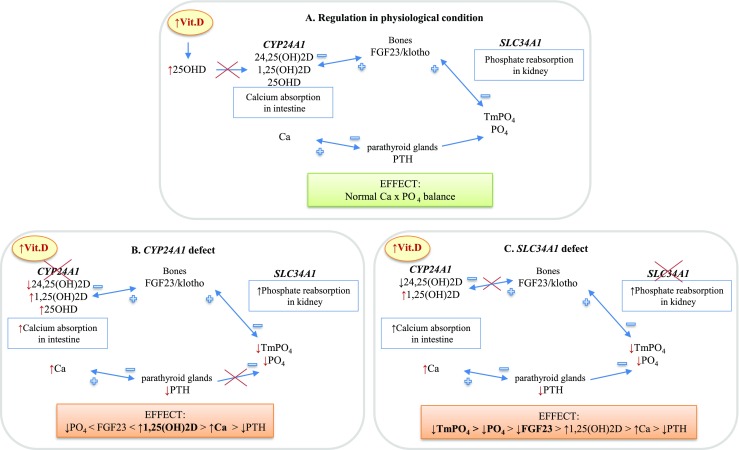
Simplified scheme of hypothetical regulation of calcium-phosphate balance in vitamin D overload status

Genetic inheritance of the IIH was confirmed only recently by the discovery of two responsible genes *CYP24A1* and *SLC34A1* (Schlingmann et al. 2011, 2016). Biallelic mutations in these genes have a completely different mechanism leading to (causing) hypercalcemia (Fig. 1). In the first *CYP24A1* defect, hypercalcemia is directly caused by impaired degradation of 1,25(OH)_2_D (Schlingmann et al. 2011). In the *SLC34A1* defect, hypercalcemia is a sequel of primary loss of phosphate in the kidney followed by downregulation of FGF-23, which in-turn leads to increased vit. D activity (Schlingmann et al. 2016).

The aim of the study was to search for pathogenic variants of *CYP24A1* and *SLC34A1* in adult persons who were shortlisted in infancy as “hypersensitive to vit. D”. The majority of them were described in childhood (Pronicka et al. 1985, 1997; Misselwitz et al. 1990).

## Patients and methods

There were two criteria for the study recruitment: (1) historical diagnosis of “vit. D hypersensitity” and (2) current adult age of the invited patients. Informed consent for DNA analysis was given by 11 patients out of 17 (64.7%) to whom a registered letter of invitation was successfully delivered.

All 11 patients included in the study were qualified as “vit. D hypersensitive” depending on the common features used by us in the 1970–1990s, as follows: (1) temporal link between appearance of hypercalcemia and administration of vit. D; (2) low PTH level (exclusion of hyperparathyroidism); (3) increased echogenicity of renal pyramids on ultrasound; (4) sustained tendency to hypercalcemia; (5) immediate response to steroid treatment, and/or low calcium diet. They presented in infancy with loss of appetite, failure to thrive, arrest in psychomotor development, constipation, and dehydration. Some patients needed several months of hospital treatment. Over childhood, the patients remained under the care of the calcium-phosphate clinic (ER) in our institute for 2–18 years (median value 14 years). Gradual normalization of clinical status and catch up in growth were observed after recovery from the episode of hypercalcemic decompensation in infancy. During the development period some of the children needed orthopedic intervention and rehabilitation due to mild dysplastic changes of hips, knees or feet. Management, at that time, included: permanent withdrawal of vit. D supply and sun protection, dietary calcium restriction, and increased fluid intake. Despite the above prophylaxis, the 25-OHD concentration maintained within high/normal limits, and laboratory data showed a tendency to hypercalcemia, hypercalciuria, and relatively low phosphate levels throughout childhood (Table 1).

**Table 1:** Clinical characteristics and molecular findings in 11 adult patients with infantile idiopathic hypercalcemia (IIH)

Patient, sex, age in years	Onset in months	Period of observation in years	Serum calcium mmol/l	Serum phosphate mmol/l	TmPO4	25-OHD ng/ml	1,25-(OH)2D pg/ml	Nucleotide change	Amino acid change
Subset with *CYP24A1* variants									
P1, F, 34	5	15 -	2.59 (M = 2.53) (2.22–3.37) *N* = 20	1.42 (M = 1.47) (1.06–1.8) *N* = 25	1.22 (M = 1.27) (0.88–1.43) *N* = 15	18.7 (M = 17.4) (13.6–25) *N* = 3	76.4 (M = 63.2) (19.1–169) *N* = 9	c.[1186C > T];[1186C > T]	p.[R396W];[R396W]
P2, F, 30	5	13	2.61 (M = 2.56) (2.54–3.17) *N* = 13	1.36 (M = 1.34) (1.09–1.85) *N* = 20	1.17 (M = 1.13) (0.74–1.61) *N* = 14	56.6 (M = 36.1) (12–186) *N* = 13	40.2 (M = 36.1) (23.8–68.3) *N* = 6	c.[428_430del];[1186C > T]	p.[E143del];[R396W]
P3, M, 28	5	18	2.71 (M = 2.61) (2.35–3.72) *N* = 54	1.48 (M = 1.56) (0.86–1.99) *N* = 41	1.29 (M = 1.31) (0.93–1.76) *N* = 29	32.5 (M = 29.9) (9.9–102.8) *N* = 23	40.5 (M = 40.1) (24.9–51.2) *N* = 5	c.[107delC];[443 T > C]	p.[P36Lfs*11];[L148P]
P4, F, 28	4.5	14	2.43 (M = 2.41) (2.06–2.91) *N* = 32	1.55 (M = 1.57) (1.09–1.89) *N* = 18	1.25 (M = 1.24) (0.99–1.58) *N* = 11	34.8 (M = 32.4) (9.3 + 73.1) *N* = 15	41.2 (M = 37.5) (12.7–84.7) *N* = 5	c.[1186C > T];[1186C > T]	p.[R396W];[R396W]
P5, M, 27	15	15	2.51 (M = 2.48) (2.38–2.72) *N* = 15	1.51 (M = 1.52) (1.31–1.71) *N* = 13	1.28 (M = 1.31) (1.11–1.45) *N* = 9	33 (M = 29.4) (10.1–95.4) *N* = 16	54.7 (M = 57.7) (28.9–74.6) *N* = 4	c.[428_430del];[964G > A]	p.[E143del];[E322K]
P6, M, 25	10	16	2.56 (M = 2.22) (2.29–3.28) *N* = 40	1.77 (M = 1.75) (1.03–2.61) *N* = 11	1.59 (M = 1.58) (0.83–2.25) *N* = 30	31.9 (M = 30.6) (4.9–90.8) *N* = 19	45.8 (M = 30.5) (2–120,5) *N* = 7	c.[1186C > T];[1226 T > C]	p.[R396W];[L409S]
P7, F, 24	3	2	2.71 (M = 2.62) (2.45–3.29) *N* = 9	1.31 (M = 1.06) (0.8–2.26) *N* = 5	1.94 *N* = 1	66.5 (M = 66.5) (57.9–75.2) *N* = 2	ND	c.[1186C > T];[1186C > T]	p.[R396W];[R396W]
P8, F, 23	4.5	18	2.72 (M = 2.68) (2.3–4.05) *N* = 27	1.3 (M = 1.32) (0.5–1.88) *N* = 23	1.17 (M = 1.09) (0.82–1.68) *N* = 13	37 (M = 36.3) (16.7–56.2) *N* = 11	74.5 (m = 80.1) (11.1–131.6) *N* = 12	c.[1186C > T];[1157 + 1G > A]	p.[R396W];[?]
P9, M, 22	4	3.5	3.08 (M = 2.85) (2.34–4.22) *N* = 30	1.52 (M = 1.7) (0.6–1.97) *N* = 17	1.75 (M = 1.72) (1.71–1.83) *N* = 3	36.9 (M = 30.9) (12.5–86.7) *N* = 7	79.9 (M = 65.6) (7.8–166.4) *N* = 3	c.[1186C > T];[1186C > T]	p.[R396W];[R396W]
Subset with *SLC34A1* variants									
P10, M, 28	12	7	2.57 (M = 2.55) (2.34–2.99) *N* = 31	1.32 (M = 1.3) (0.92–1.94) *N* = 28	1.13 (M = 1.15) (0.72–1.52) *N* = 21	21.2 (M = 18.9) (7–47.5) *N* = 14	40.9 (M = 36.6) (21.6–82.1) *N* = 9	c.[272_292del];[464 T > C]	p.[V91_A97del];[L155P]
P11, F, 26	2.5	7	2.58 (M = 2.55) (2.22–3.1) *N* = 49	1.54 (M = 1.55) (1.13–1.84) *N* = 31	1.38 (M = 1.41) (1.06–1.65) *N* = 16	35.4 (M = 34.3) (6.8–139) *N* = 21	45.4 (M = 49.3) (9.4–103.5) *N* = 10	c.[1425_1426del];[?]	p.[C476Sfs*128];[?]

*Parathyroid hormone measured sporadically using various methods was always very low; M, median; N, number of measurements; ND, not determined; F, female; M, male; mutation numbering was based on the cDNA sequence (human *CYP24A1*, GenBank NM_000782.4, NP_000773.2; human *SLC34A1*, GenBank NM_003052.4, NP_003043.3) according to the guidelines of Human Genome Variation Society v2.0 Nomenclature (HGVS, www.hgvs.org/mutnomen)

In nine patients Sanger sequencing analysis was carried out in Muenster according to the protocol described previously (Schlingmann et al. 2011, 2016). In two patients (P7 and P9) next-generation sequencing (NGS) was conducted. NGS was performed on a HiSeq 1500 using TruSight One Sequencing Panel (Illumina) according to the manufacturer’s instructions. Generated reads were aligned to the hg19 reference human genome. The detected variants were annotated using Annovar (Wang et al. 2010) and converted to MS Access format for final manual analyses. Bioinformatics analysis was performed as previously described (Ciara et al. 2016). Alignments were viewed with Integrative Genomics Viewer v.2.2.79 (Robinson et al. 2011). Mutation numbering was based on the cDNA sequence (human *CYP24A1*, GenBank NM_000782.4; human *SLC34A1*, GenBank NM_003052.4) according to the guidelines of Human Genome Variation Society (HGVS, www.hgvs.org/mutnomen).

Molecular analysis was performed after obtaining written informed consent.

## Results and discussion

At the study beginning, all patients were working or studying. They were in very good health and without complaints. Mean weight, height, and body mass index (BMI) were respectively 180.7 ± 10.1 cm, 83.4 ± 31.6 kg, and 25 ± 7 for males and 164.7 ± 2.6 cm, 63.7 ± 9.1 kg, and 23.5 ± 3.8 for females. Three patients were under the control of nephrologist in the place of residence, due to sustained nephrocalcinosis (stable from infancy). Urolithiasis and progressive renal failure, reported in IIH (Molin et al. 2015) did not occur in any of the patients included in molecular study. Serum creatinine concentration measured in two patients was at bordeline of the control values (1.29, 1.13 mg/dl).

The results of molecular studies are shown in Table 1. In all 11 adults, the historical diagnosis of IIH was confirmed at the molecular level with 100% compliance. We detected pathogenic variants in one of two genes studied, *CYP24A1* in nine patients and *SLC34A1* in two remaining ones. The latter subset was already included in the original publication (Schlingmann et al. 2016).

In *CYP24A1*, two novel variants c.107del (p.P36Lfs*11) and c.1157 + 1G > A (p.?) as well as five known variants [c.428_430del (p.E143del), 443 T > C (p.L148P), c.964G > A (p.E322K), c.1186C > T (p.R396W), c.1226 T > C (p.L409S)] were identified in homozygous or compound heterozygous state (Table 1). Missense variant p.R396W occurred in seven of nine *CYP24A1* mutated persons (11/18 alleles; four homozygotes and three compound heterozygotes). Previously this variant, found in four of 1024 control alleles, was annotated as putative polymorphism; however, it finally concluded as pathogenic (Schlingmann et al. 2011).

Four p.R396W alleles were found in 588 samples (1176 alleles) from general Polish population. Based on this data the frequency of p.R396W allele was calculated as 0.0034 (0.34%; 1:294). Using the Hardy-Weinberg’s law the frequency of p.R396W carriers in the Polish population was estimated as 0.0068 (0.68%; 1:147). Taking into account that p.R396W make up approximately 61.1% of *CYP24A1* alleles, overall carrier frequency for all mutation in *CYP24A1* was calculated as 1.11% (1:90). Thus, the expected incidence of *CYP24A1* deficient IIH in Poland was estimated as 1:32,465 births (with 0.95 confidence interval). A thousand potentially affected *CYP24A1* deficient persons may be living in the country.

The study group of IIH, at start considered genetically homogeneous, turned out to be divided in two. Laboratory data was re-analyzed in two subsets of patients, with *CYP24A1* and of *SLC34A1* defects. Age of onset, total/daily vitamin D dose, duration of hypercalcemia, prednisone treatment, milk free diet, calcium/phosphate concentrations and excretion, 25OHD, 1,25(OH)_2_D, and PTH levels were assessed separately (Table 1). We were not able to identify any unequivocal feature useful to distinguish *CYP24A1* from *SLC34A1* defect at presentation of hypercalcemia in infancy. Only two potentially important differences have to be mentioned: (1) the previously reported finding that 25OHD concentration in IIH probands, carriers of two mutated variants, was twice as high as in their parents, carriers of a single mutated allele (Rowińska et al. 1998), was observed only in the families with *CYP24A1* defect, (2) kidney stones were found in one father of an IIH proband (Rowińska et al. 1998) with *SLC34A1* defect. Unfortunately those old findings are anecdotal (data no longer available) and have to be verified in the future study.

Despite the fact that, in the light of present knowledge, our historical diagnosis of “hypersensitivity to vitamin D” is correct for both, the *CYP24A1* and the *SLC34A1* defects, the identical treatment options may require a revision. We may speculate, e.g., that the long-term calcium restriction and vit. D avoidance might be incorrect and possibly harmful for the patients with *SLC34A1* defect (P10 and P11). Alternative treatment with phosphates supplementation should be considered and evaluated. By the way, increased phosphate intake was recommended constantly for our patients with IIH, and was even tested in a short experiment (Rowińska et al. 1998), however no differences in response to phosphates between individual patients were observed and no conclusions were then attained.

This lack of laboratory difference is likely due to strict control of the calcium-phosphate homeostasis to avoid the negative effects of vitamin D excess on concentration of both calcium and phosphate during mineralization. In such a situation the optimal condition for mineralization requires feedback reaction in the direction opposite to primary genetic defect (e.g., calcium absorption in *SLC34A1* and phosphate re-absorption in *CYP24A1*). As a result, the final effect of elevated 1,25(OH)_2_D is similar in both *SLC34A1* and *CYP24A1* defects (Fig. 1). It seems obvious, however, that counteracting the consequences of both defects may be different; hence, the need to establish the molecular background of IIH is crucial, not only in infants but also in adults. Our study indicates that the IIH defect is relatively frequent, and if not detected, is potentially dangerous also for adults.

The discovery of the association between IIH and the mutations in *SLC34A1* and *CYP24A1* genes opened a new research area. Several questions are under investigation, among them:

1. Are *CYP24A1* and *SLC34A1* defects neutral for health if vit. D is not excessively supplemented?
2. Whether and how *CYP24A1* and *SLC34A1* mutations contribute to modifying bone growth and calcification; are they involved in nephrolithiasis development?
3. Whether benefits of long-term calcium/vit. D restriction in IIH infants outweigh adverse events in case of *CYP24A1* and *SLC34A1* mutations?
4. What is the actual prevalence of *CYP24A1* and *SLC34A1* mutations and what is the risk throughout life of being a carrier of biallelic mutations in these genes?
5. How often do these defects in the population occur, and if indeed there are any ethnic differences?
6. Whether and on what basis can *CYP24A1* and *SLC34A1* mutations in these defects be distinguished without molecular testing (e.g, measure of 24OHD)?
